# Genome-Wide Identification and Expression Analysis of the WOX Family Reveals Potential Roles in Stem Development of *Euphorbia hirta*

**DOI:** 10.3390/plants15030509

**Published:** 2026-02-06

**Authors:** Qianyi Lyu, Shutong Chen, Xin Wang, Yuan Yuan, Hongrui Zhang, Wanqi Liang, Han Cheng, Zhi Deng

**Affiliations:** 1 National Key Laboratory for Tropical Crop Breeding, Ministry of Agriculture and Rural Affairs Key Laboratory of Biology and Genetic Resources of Rubber Tree, Rubber Research Institute, Chinese Academy of Tropical Agricultural Sciences, Haikou 571101, China; sjtu-lqy@sjtu.edu.cn (Q.L.);; 2 Yazhou Bay Institute of Deepsea Science and Technology, Shanghai Jiao Tong University, Sanya 572024, China; 3 Sanya Research Institute, Chinese Academy of Tropical Agricultural Sciences, Sanya 572025, China; 4 State Key Laboratory Incubation Base for Cultivation and Physiology of Tropical Crops, Rubber Research Institute, Haikou 571100, China; 5 College of Plant Science and Technology, Huazhong Agricultural University, Wuhan 430070, China

**Keywords:** WOX, *Euphorbia hirta*, transcription factors, stem development, expression pattern

## Abstract

The homeobox transcription factor (TF) superfamily includes the WUSCHEL-RELATED HOMEOBOX (WOX) family, which plays a critical role in adaptive plant growth. Specifically, WOX regulates stem growth in plants, with stems serving as the structural framework for laticifers in *Euphorbia hirta*. However, the number of *WOX* gene family members in the *E. hirta* genome has not been reported. In this study, we identified 14 *EhWOX* genes in *E. hirta* and characterized their physicochemical properties, chromosomal locations, phylogenetic relationships, conserved motifs, gene structures, promoter *cis* elements, gene ontology (GO) enrichment, tissue-specific expression patterns, and subcellular localization. Chromosomal mapping indicated their distribution across nine chromosomes. Phylogenetic analysis classified these genes into three evolutionary clades. Promoter *cis*-element analysis identified abundant light-responsive, hormone-responsive, and stress-responsive elements. GO enrichment suggested their broad involvement in diverse biological processes. Additionally, RNA-seq revealed high expression levels of *EhWOX4*-*6* and *EhWOX14* in stems. Furthermore, RT-qPCR confirmed tissue-specific expression in stems. Moreover, experimental evidence confirmed the subcellular localization and autoactivation capability of some WOX proteins that may be involved in stem development. Overall, this study provides a comprehensive characterization of the candidate *EhWOX* genes and provides a foundational resource for future functional investigations into their possible roles in stem and laticifer biology.

## 1. Introduction

Plants possess numerous TF families that play critical roles in regulating diverse cellular metabolic activities and signaling pathways during stress responses and developmental processes. Among TF families, the WOX TF family, first identified in *Arabidopsis thaliana* in 1996, has been demonstrated to be particularly crucial for shoot meristem maintenance and floral organ development [1]. The *WOX* genes represent a plant-specific family of TFs belonging to the homeodomain (HD) superfamily, currently classified into 14 distinct subfamilies based on phylogenetic relationships [2,3,4]. Characteristically, WOX proteins contain a highly conserved 60–66 amino acid homeodomain that forms a helix–turn–helix structure, with the N-terminal HD domain enabling high-affinity monomeric binding to specific DNA sequences [5,6]. Phylogenetic analyses have consistently demonstrated that the *WOX* gene family is evolutionarily classified into three major clades, including the ancient clade (AC), intermediate clade (IC), and modern/WUS clade (WC) [7,8]. Comparative genomic studies reveal that the AC originated in embryophytes and chlorophytes [9], while the IC emerged in lycophytes and seed plants and the WC evolved from the common ancestor shared by spermatophytes and bryophytes [3,10]. This tripartite phylogenetic division reflects the progressive evolutionary trajectory and functional diversification of *WOX* genes across land plants.

*E. hirta*, native to Central America and later introduced to Southeast Asia, is now widely distributed across tropical and subtropical regions worldwide. Commonly known as asthma weed, snakeweed, or ara tanah, it belongs to the Euphorbiaceae family and the Euphorbia genus [11]. As a medicinal herb, the plant is rich in bioactive compounds, including polyphenolic flavonoids, tannins, terpenoids, and triterpenoids [12,13,14], which contribute to its unique pharmacological properties. *E. hirta* exhibits strong reproductive capacity, thriving in grassy and shrubby hillsides, particularly in sandy soils [15]. Furthermore, this annual herbaceous species typically attains a height of 15–50 cm and exhibits either erect or procumbent stems containing laticifers that produce latex. These morphological characteristics make it an ideal model system for investigating stem and laticifer development.

Indeed, genetic and molecular biological studies on plant *WOX* genes have demonstrated their crucial roles in various developmental processes [8,16,17]. Since they play vital functions in shoot and floral development [1], research has extensively characterized *WOX* gene functions in developmental regulation across multiple plant species. During plant growth and development, the plant stem functions as an essential junction connecting roots and leaves, performing multiple pivotal functions, including mechanical support, nutrient transport, storage, reproduction, and photosynthesis. Research has established that in stem development, the *WOX4* gene forms a signaling hub with *BES1* to regulate the proliferation and differentiation of vascular cambium cells in response to both hormonal and environmental signals, and *wox4* mutants exhibit normal xylem differentiation but impaired cambium cell proliferation [18]. In cotton, knockout of *GhWOX4* reduces cambium width and division activity, consequently suppressing secondary growth [19]. In hybrid aspen (*Populus tremula* × *tremuloides*), *WOX4* modulates both cambium cell identity and mitotic activity [20], while in Scots pine (*Pinus sylvestris*), *PsWOX4* expression peaks during active cambium proliferation periods, with the highest cambium cell layers observed in mature trees (63-year-old) [21]. Additionally, further investigations reveal that *AtWOX5*, *AtWOX8*/*9*, and *AtWOX14* play crucial roles in stem development [22]. In rubber tree, previous studies have suggested that the CLAVATA-MAPK-WOX signaling pathway may be involved in regulating secondary laticifer differentiation in *Hevea brasiliensis* [23]. Meanwhile, research has characterized *WOX* genes in *H. brasiliensis*, *Jatropha curcas*, *Manihot esculenta*, and *Ricinus communis* [24]. These studies demonstrate that *WOX* genes have been well studied in across multiple plant species, whereas their functions in the Euphorbiaceae family, which diverged from Arabidopsis approximately 100 million years ago [25], remain largely unexplored and the functions of *WOX* genes in *E. hirta* remain unreported.

Despite the established roles of WOX genes in stem development and the economic and medicinal importance of *E. hirta*, a comprehensive characterization of the WOX family in this species is lacking, and its potential involvement in stem development remains unexplored. In this study, we systematically identified 14 *EhWOX* genes in *E. hirta*. These genes were subjected to comprehensive characterization through multiple approaches, such as bioinformatics analyses, including phylogenetic tree construction, chromosomal localization, conserved motif and gene structure analysis, synteny analysis, cis-acting element prediction, GO enrichment, and regulatory network prediction. The expression patterns of *EhWOX* genes in various tissues were analyzed by RNA-seq; then, expression in stems and leaves was further validated by RT-qPCR. Additionally, functional validation was achieved through subcellular localization in *Nicotiana Benthamian* and transcriptional autoactivation in yeast. This study presents the first systematic characterization and expression profiling of *WOX* genes and provides a foundation for functional validation of candidate WOX proteins in *E. hirta.*

## 2. Results

### 2.1. Identification of EhWOXs and Prediction of Physicochemical Properties

The hidden Markov model (HMM) was employed to identify *EhWOX* homeodomains, followed by local BLASTP searches using *A. thaliana WOX* genes as a reference. A total of 14 candidate *EhWOX* genes were identified based on the genome of *E. hirta* (Supplementary Table S1). Bioinformatic analysis predicted that their amino acid (AA) lengths ranged from 170 to 385 residues, with molecular weights (MW) between 19.9 and 42.36 kDa. Further predicted characterization showed that their theoretical isoelectric points (pI) varied from 5.15 to 9.83. All EhWOX proteins exhibited instability indices exceeding 40 (from 43.09 to 74.23), suggesting their inherent instability. The aliphatic index ranged from 51.75 to 73.86, while the values of the grand average of hydropathicity (GRAVY) were all negative (−0.475 to −1.057) (Table 1), indicating strong hydrophilicity. Additionally, subcellular localization predictions placed EhWOX proteins in the nucleus.

### 2.2. WOX Numbers Across Species and Chromosomal Locations of EhWOXs

**Figure 1:**
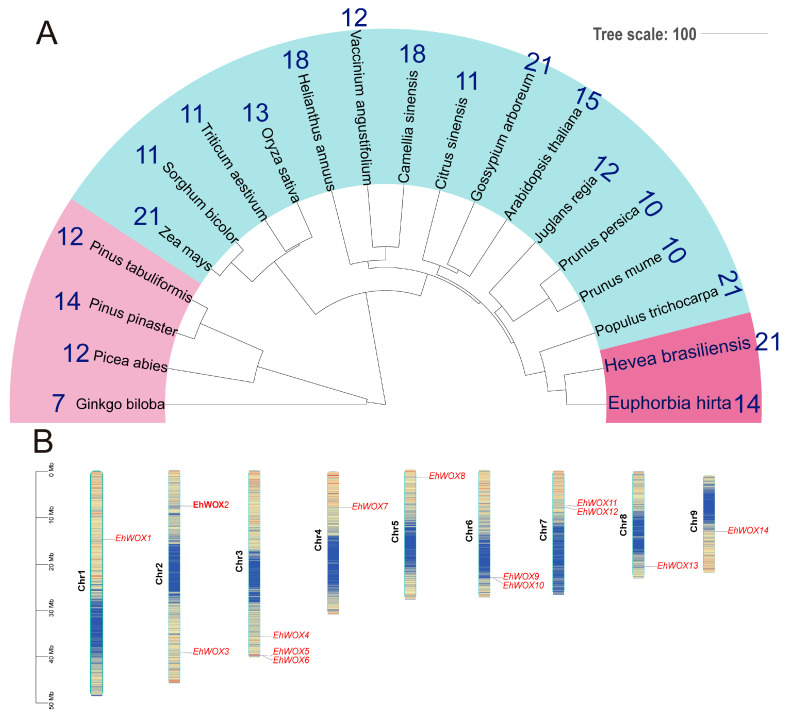
Phylogenetic analysis of *E. hirta*, *WOX* gene numbers across multiple species and chromosomal distribution of *EhWOX* genes. (**A**). Time-calibrated phylogenetic tree of *E. hirta* and 19 plant species; the outermost numbers indicate the *WOX* gene count for each species, and the time scale is 100 MYA (million years ago). (**B**). Chromosomal distribution of *EhWOX* genes. A total of 14 *EhWOX* genes were randomly distributed across nine chromosomes, with gradient colors (blue to orange) indicating increasing gene density. The scale is in megabases (Mb).

To compare *WOX* numbers across species, a phylogenetic tree of 20 plant species was constructed, and the number of *WOX* genes in each species was quantified (Figure 1A) [8]. The divergence time tree revealed that *E. hirta* and *H. brasiliensis* clustered within the same clade, indicating their close phylogenetic relationship. However, differences were observed among the *WOX* gene family members, with 14 *EhWOX* genes identified in *E. hirta* and 21 in *H. brasiliensis*, which may result from gene evolution driven by functional demands [26,27]. Notably, this higher number of *WOX* genes in *H. brasiliensis* was comparable to that in *Zea mays* and *Gossypium arboreum*. Additionally, chromosomal localization analysis demonstrated that the 14 *EhWOX* genes were randomly distributed across nine chromosomes in *E. hirta* (Figure 1B). Chromosome three contained the highest number (three genes), while chromosomes two, six, and seven each harbored two *EhWOX* genes and the remaining chromosomes contained one *EhWOX* gene, respectively.

### 2.3. Phylogenetic Analysis, Conserved Domains and Gene Structural Analysis of EhWOXs

**Figure 2:**
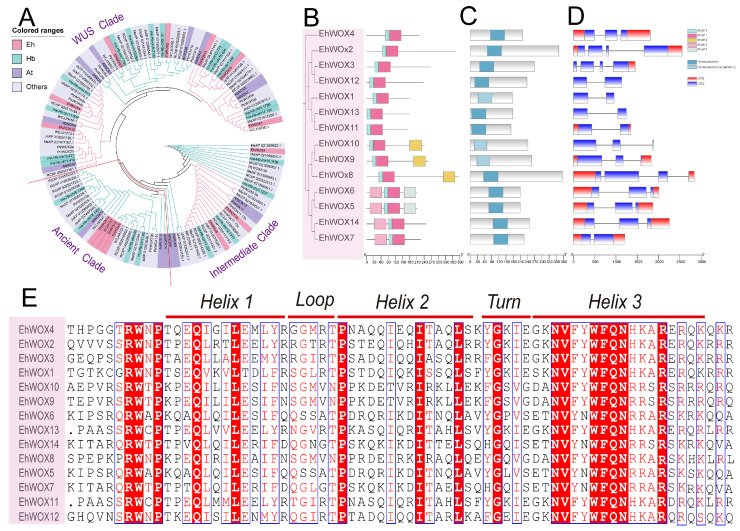
Phylogenetic and structural characterization of the EhWOX family. (**A**) Phylogenetic tree of WOX proteins from eight species (Eh: *E. hirta*; WCJ: *E. lathyrism*; Ja: *J. curcas*; Me: *M. esculenta*; Rc: *R. communis*; rna-Hb: *H. brasiliensis*; Ptr: *P. tomentosa*; At: *A. thaliana*). The ML tree was constructed with bootstrap values using 5000 replicates, proteins from different species are color-coded, and the red lines divide the phylogenetic tree into three clades. (**B**) Distribution of conserved motifs in EhWOX proteins. Five predicted motifs are represented by distinct colored boxes. (**C**). Each blue box marks the homeodomain of an EhWOX protein. (**D**) Gene structure of *EhWOX* members. Exons (blue boxes), introns (lines), and UTRs (red boxes) are displayed proportionally to genomic sequence lengths. (**E**) Multiple sequence alignment of the homeodomain region in EhWOX proteins. Conserved residues are highlighted by color intensity according to identity levels, blue boxes indicate conserved amino acid regions, with white letters on red background representing the most conserved amino acids. Key structural features (Helix 1, Loop, Helix 2, Turn, and Helix 3) are annotated above the alignment. Red lines indicate the ranges of key structural features.

Phylogenetic reconstruction was conducted using the neighbor-joining method with amino acid sequences of WOXs from *E. hirta*, *H. brasiliensis*, *A. thaliana*, and other species. Consistent with the phylogenetic clade of *A. thaliana* AtWOXs [28], the EhWOX proteins were classified into three distinct subfamilies (Figure 2A). Each subfamily contained WOX members from both *A. thaliana* and *H. brasiliensis*. Notably, the EhWOX proteins formed distinct branches that were phylogenetically distant from their orthologs in *H. brasiliensis* and *A. thaliana*, suggesting potential functional divergence during the evolution of *E. hirta*. Conserved motif and gene structure analyses were performed to investigate the evolutionary relationships among the 14 *EhWOX* genes. MEME analysis identified five conserved motifs in the EhWOX family (Figure 2B). Notably, motif 1 and motif 3 were present in all 14 *EhWOX* genes, with motif 1 consistently positioned to the right of motif 3, and seven genes (*EhWOX4*, *EhWOX2*, *EhWOX3*, *EhWOX12*, *EhWOX1*, *EhWOX13*, and *EhWOX11*) only contained motifs 1 and 3 of five motifs. Additional motif analysis revealed that four genes (*EhWOX6*, *EhWOX5*, *EhWOX14*, and *EhWOX7*) possessed motif 2, while three genes (*EhWOX10*, *EhWOX9*, and *EhWOX8*) contained motif 4. Moreover, only two genes (*EhWOX6* and *EhWOX5*) exhibited motif 5, suggesting potential functional diversification among *EhWOX* members. NCBI-CDD domain prediction showed that all 14 genes belong to the Homeodomain superfamily, with 11 possessing canonical homeodomains (Homeodomain family) and 3 (EhWOX10, EhWOX9, and EhWOX1) showing structural variations (Figure 2C). Gene structure analysis revealed that *EhWOX* genes contained one to three introns (Figure 2D), while protein sequence alignment revealed that the Helices, Loops, and Turns within the conserved homeodomain of the 14 EhWOX proteins exhibited highly similar amino acid distributions (Figure 2E). These findings suggest that this gene family is highly conserved during evolution and may play a crucial role in plant growth and development.

### 2.4. Duplication Events and Evolutionary Characterization of EhWOXs

**Figure 3:**
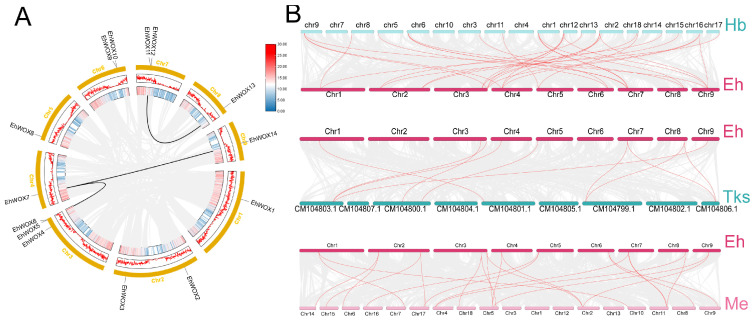
Collinearity analysis of the *EhWOX* gene family. (**A**) Analysis of tandem replication of *EhWOX* genes in the genome of *E. hirta*. The three concentric rectangles, arranged from the outermost to the innermost, correspond to chromosomes, GC content, and gene density. Chromosomes 1~9 are represented by rectangles. Lines and histograms along the rectangle represent the gene density on the chromosome. The gray lines represent the isolinear blocks in the genome of *E. hirta*, while the black lines between chromosomes represent gene pairs with fragment repeats. (**B**) Comparative synteny analysis among *H. brasiliensis* (Hb), *T. kok-saghyz* (Tks), and *M. esculenta* (Me). Gray lines in the background highlight collinear blocks within *E. hirta* and other plant genomes, while collinear *EhWOX* gene pairs are linked with red lines.

Gene duplication events include whole-genome duplication (WGD), tandem duplication, segmental duplication, and dispersed duplication, which contribute to the generation of homologous genes with sequence similarity. Tandem duplication events are defined as chromosomal regions containing two or more genes within a 200 kb genomic interval; they are particularly important for evolutionary adaptation, gene regulation, and genome stability [29]. Using the MCScanX algorithm, we identified diverse duplication types among *EhWOX* genes (Supplementary Table S2). Specifically, *EhWOX6* originated from proximal duplication, while *EhWOX10* and *EhWOX9* resulted from tandem duplication. Five genes (*EhWOX13*, *EhWOX11*, *EhWOX5*, *EhWOX14*, and *EhWOX7*) were derived from WGD or segmental duplication events, with the remaining six genes classified as dispersed duplications, demonstrating the heterogeneous origins of *EhWOX* genes. Collinearity analysis revealed three syntenic gene pairs distributed across five chromosomes (Figure 3A). Notably, *EhWOX7* formed two syntenic pairs and clustered with *EhWOX14* in the phylogenetic tree, despite their localization to different chromosomes (Chr4 and Chr9). Additionally, comparative synteny analysis with *H. brasiliensis*, *Taraxacum kok-saghyz*, and *M. esculenta* demonstrated distinct evolutionary patterns (Figure 3B). *E. hirta* exhibited 28, 10, and 23 syntenic gene pairs with these species. The higher number of syntenic pairs with *H. brasiliensis* and *M. esculenta* compared to *T. kok-saghyz* suggests stronger evolutionary conservation between these species. These findings indicate that *WOX* genes have undergone multiple duplication events during speciation while maintaining functional conservation, underscoring their essential roles in plant growth and development.

### 2.5. Characterization of Cis-Acting Elements in Promoters of EhWOXs

**Figure 4:**
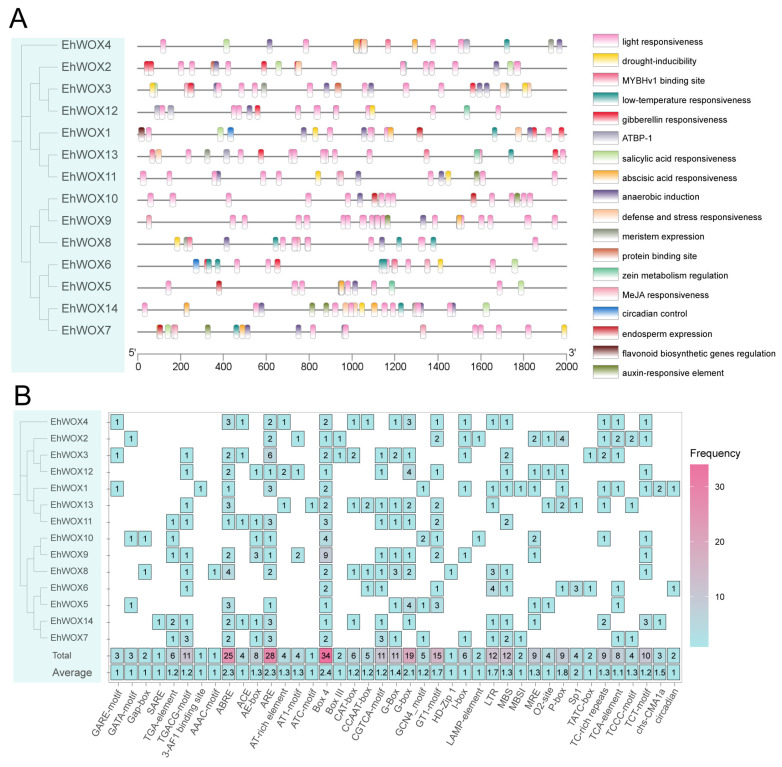
Cis-acting elements in the promoters of the *EhWOX* family. (**A**) Distribution of cis-acting elements in *EhWOX* family promoters. Different colored blocks represent distinct types of cis-acting elements. (**B**) Numbers of cis-acting elements in *EhWOX* gene family promoters. The color gradient from blue to red indicates increasing numbers of elements.

To elucidate the potential regulatory relationship of *EhWOX* genes, we analyzed cis-acting elements within the 2000 bp upstream regions of all 14 *EhWOX* genes. As a result, a total of 18 distinct cis-regulatory elements were predicted (Figure 4). The promoter regions were particularly enriched with light-responsive, hormone-related, and stress-responsive elements. Light-responsive elements were the most abundant category, with each *EhWOX* gene containing 5–15 Light-responsive elements (Box 4, G-Box, and GT1 motif) and *EhWOX9* showing the maximum number (15 elements). Hormone-responsive elements were also widely distributed, including abscisic acid-responsive ABRE elements and JA-responsive CGTCA/TGACG motifs. Notably, five genes (*EhWOX4*, *EhWOX2*, *EhWOX1*, *EhWOX10*, and *EhWOX5*) lacked JA-responsive elements, while the remaining genes contained varying numbers of JA-responsive motifs. Only two genes (*EhWOX13* and *EhWOX6*) lacked ARE elements. The predicted cis-element profile suggests that *EhWOX* genes are likely involved in diverse physiological processes, including light signaling, hormonal regulation, and stress responses, highlighting their potential roles in adaptive plant growth and development.

### 2.6. GO and Regulatory Network Predictions of EhWOXs

**Figure 5:**
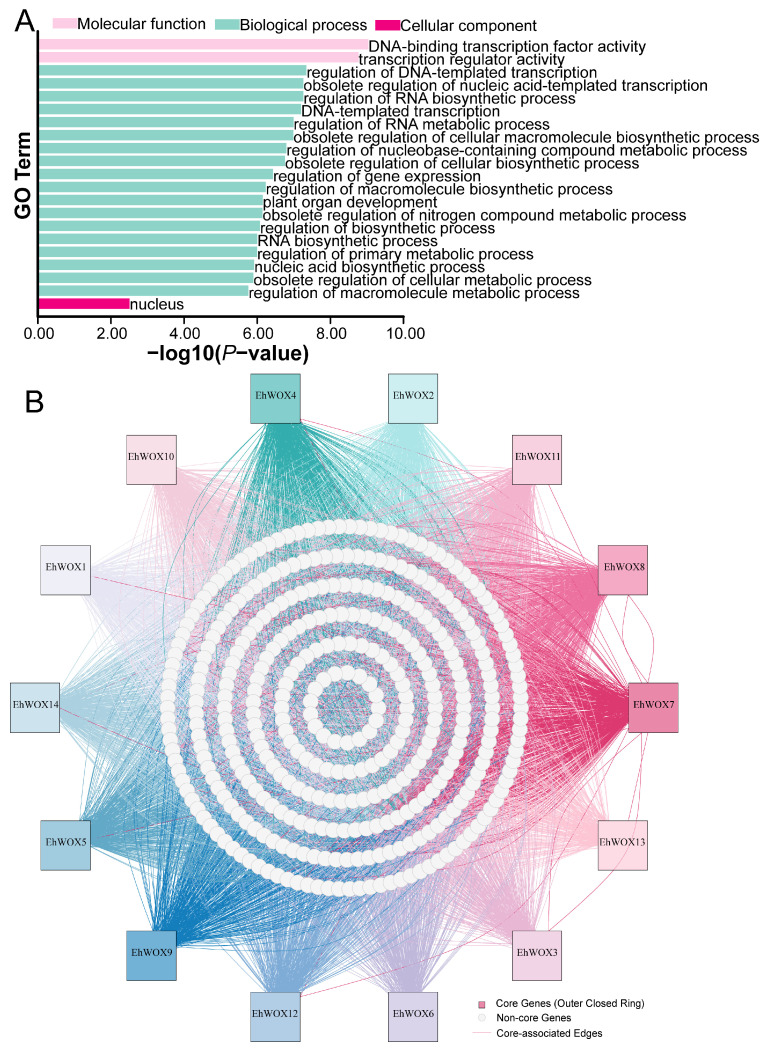
Functional annotation and regulatory network prediction of *EhWOX* genes. (**A**) GO enrichment analysis of *EhWOX* genes. The top 20 significantly enriched terms in the “biological process” category are displayed. (**B**) Predicted TF binding network for *EhWOX* genes in *E. hirta*. The outer 14 squares represent the *EhWOX* genes, while the inner 6 white concentric circles indicate predicted target genes with “Excellent” and “Fair” confidence scores.

Studies across plant species have demonstrated the involvement of *WOX* gene family members in diverse biological processes. In this study, the predicted result revealed significant enrichment in the GO terms of 45 biological process (Supplementary Table S3). Figure 5A displays the top 18 most significantly enriched biological process terms, 2 molecular function terms, and 1 cellular component term. The five most significantly enriched terms comprised regulation of DNA-templated transcription (GO:0006355), obsolete regulation of nucleic acid-templated transcription (GO:1903506), regulation of RNA biosynthetic process (GO:2001141), DNA-templated transcription (GO:0006351), and regulation of RNA metabolic process (GO:0051252).These predicted analyses support the canonical role of WOX family members as TFs that regulate plant growth and development by binding to promoter regions of target genes and directly modulating DNA/RNA-related activities. In addition, based on the complete sequences of *E. hirta*, we identified transcription factor binding motifs across all sequences, obtaining a total of 10,155 potential binding motif pairs involving 14 EhWOX TFs through the FIMO tool, encompassing 630 genes, including these 14 *EhWOX* genes (Supplementary Table S4). A total of 362 potential target genes with prediction scores of “Excellent” or “Fair” were displayed (Figure 5B). Among these, *EhWOX4*, *EhWOX14*, and *EhWOX12* exhibited the highest predicted motif binding counts (465, 461, and 413 pairs, respectively), while *EhWOX6* showed the lowest (259 pairs). These predicted results demonstrate that *EhWOX* TFs exhibit differential DNA-binding properties in *E. hirta*, suggesting their potential involvement in plant physiological processes through varying regulatory intensities on target genes. The construction of the *EhWOX* transcriptional regulatory network provides foundational data for subsequent research.

### 2.7. Expression Patterns of WOX Genes in E. hirta Tissues

**Figure 6:**
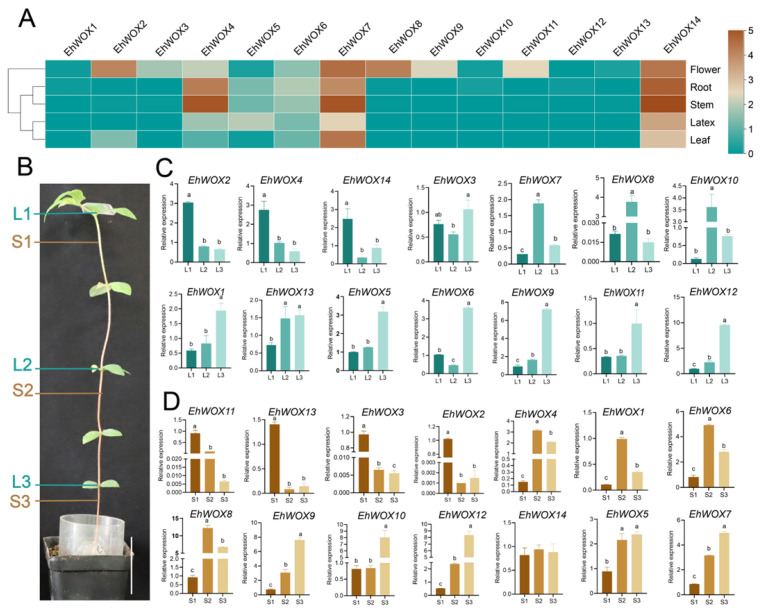
Expression patterns of *EhWOX* genes in *E. Hirta* tissues. (**A**) Heatmap showing expression levels (FPKM values) of 14 *EhWOX* genes across five tissues (flower, root, stem, leaf, and latex). (**B**) Sampling sites for RT-qPCR analysis in leaves and stems (L: leaf; S: stem; bar: 5 cm). (**C**,**D**) Tissue-specific expression profiles of the 14 *EhWOX* genes in leaves and stems from three distinct developmental positions of *E. hirta*. One-way ANOVA and Tukey’s multiple comparison test were used to evaluate the statistical significance. Different letters indicate significance at *p* < 0.05. The error bar represents the standard deviation of three biological replicates.

To investigate the potential roles of *EhWOX* genes in *E. hirta*, we analyzed their expression patterns across five tissues (flower, latex, leaf, root, and stem) using RNA-seq data. Heatmap analysis revealed distinct tissue-specific expression profiles among the 14 *EhWOX* genes (Figure 6A, Supplementary Table S5). Notably, *EhWOX7* and *EhWOX14* showed consistently high expression across all examined tissues, with particularly high expression in stems, roots, and flowers. The *EhWOX4*, *EhWOX5*, and *EhWOX6* genes also exhibited stem-predominant expression (S2 and S3), with these genes predicted to play crucial roles in stem development and maintenance. Furthermore, *EhWOX14* and *EhWOX7* displayed relatively high expression in latex, implying their potential involvement in latex biosynthesis. To validate these expression patterns, RT-qPCR was performed on three distinct segments of leaves (L1–L3) and stems (S1–S3) from seedlings (Figure 6B–D). Leaf RT-qPCR showed that three genes were highly expressed in L1, with 7 genes highly expressed in L2, and 9 genes highly expressed in L3 (Figure 6C). Additionally, the result revealed four genes with high expression in S1, eight genes with high expression in S2, and eight genes with high expression in S3(Figure 6D). Importantly, the stem-specific expression patterns of *EhWOX4*, *EhWOX7*, *EhWOX14*, *EhWOX5* and *EhWOX6* observed in RNA-seq data were confirmed by RT-qPCR. The RNA-seq results demonstrate that *EhWOX* genes exhibit tissue-specific expression patterns, and further RT-qPCR analysis of *WOX* gene expression revealed that several genes may be involved in stem development.

### 2.8. Subcellular Localization and Autoactivation of EhWOXs

**Figure 7:**
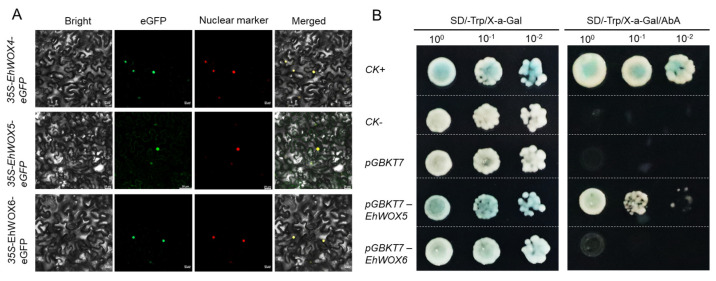
Subcellular localization and autoactivation of EhWOXs. (**A**) Subcellular localization of EhWOX proteins. Transient expression in *N. benthamiana* leaves showing subcellular localization of three EhWOX proteins (EhWOX4, EhWOX5, and EhWOX6). Constructs were generated by homologous recombination of target genes into the *35S-pCAMBIA2300-eGFP* vector, and subsequently co-infiltrated with the nuclear localization marker gene (*35S-OsWRKY70-RFP*) into tobacco leaves. Confocal microscopy images show bright-field, GFP, RFP, and merged signals. Scale bar = 20 μm. (**B**) Autoactivation verification of EhWOX proteins. Transcriptional autoactivation assay in yeast. EhWOX5 and EhWOX6 TFs were cloned into the *pGBKT7* vector and tested for autoactivation activity, respectively. Both showed positive results, as evidenced by growth on selective medium (-Trp/-X-α-Gal) and (-Trp/-AbA/-X-α-Gal) (AbA: 200 ng/mL).

Bioinformatic analysis predicted nuclear localization for 14 *EhWOX* TFs (Table 1). To validate these predictions, GFP fusion vectors (*pCAMBIA2300-EhWOX4-eGFP*, *pCAMBIA2300-EhWOX5-eGFP*, and *pCAMBIA2300-EhWOX6-eGFP*) were constructed and transiently expressed in *Nicotiana benthamiana*. Microscopic detection demonstrated that three *EhWOX-GFP* fusion proteins exhibited nuclear localization, which colocalized with the *OsWRKY70-RFP* nuclear marker (Figure 7A). These results confirm the computational predictions and provide experimental evidence supporting the TF function of EhWOX proteins. Furthermore, sequence alignment of EhWOX5 and EhWOX6 revealed only one amino acid difference in the homeodomain (EhWOX5: Leu; EhWOX6: Pro) and a total of 11 amino acid differences across their full-length sequences (Figure 2E, Supplementary Table S1). Therefore, to further investigate their functional differences, autoactivation validation was first performed. The CDSs of EhWOX5 and EhWOX6 were cloned into the *pGBKT7* vector to generate recombinant *pGBKT7-EhWOX* vectors. Then, the yeast colonies were dropped onto SD/-Trp/-X-α-gal medium to assess their autoactivation capability (Figure 7B). Additionally, the addition of AbA (200 ng/mL) as an inhibitor, combined with X-α-gal staining analysis, revealed that 200 ng/mL AbA significantly suppressed the growth of yeast colonies (Figure 7B). Collectively, the results confirm that these two proteins are autoactivating.

## 3. Discussion

The regulation of plant growth and development involves numerous TFs, among which the *WOX* gene family represents one of the most conserved TF families in plants. With advances in genome sequencing technologies, *WOX* gene families have been identified and characterized in various plant species (Figure 1A), including *H. brasiliensis*, *J. curcas*, *M. esculenta*, and *R. communis* within the Euphorbiaceae family [24]. However, systematic characterization of the *EhWOX* family in *E. hirta* has not been reported. In this study, through a combination of local BLASTP, HMMER, CDD, and conserved motif analyses, we identified 14 *EhWOX* genes distributed across nine chromosomes of *E. hirta* (Table 1, Figure 1B, Supplementary Table S1). Subsequently, a series of bioinformatics analyses, including assessments of protein physicochemical properties, phylogenetic tree construction, gene structure analysis, synteny analysis, cis-regulatory element identification, and GO and transcriptional regulatory network analysis, was performed. Additionally, tissues expression profiling was conducted, followed by validation of stem and leaf expression patterns through RT-qPCR. Moreover, subcellular localization and autoactivation assays were performed. These comprehensive analyses establish a foundational framework for further functional characterization of the *EhWOX* family.

Studies have demonstrated that the evolution of the *WOX* family is closely associated with morphological innovations during plant speciation [28,30,31]. In this study, we conducted temporal evolutionary analysis of *E. hirta*, *H. brasiliensis*, *A. thaliana*, and 17 other species and quantified *WOX* gene family members in each species [8] (Figure 1A). Notably, *H. brasiliensis* contains 21 *WOX* genes, while *E. hirta* has 14 *WOX* genes. In *E. hirta*, only three syntenic gene pairs were identified, whereas 28 homologous pairs were found between *E. hirta* and rubber tree (Figure 3). Studies have shown that *H. brasiliensis* underwent a recent WGD event, resulting in more retained duplicate gene copies in the rubber tree genome [32]. Moreover, as a perennial latex-producing plant, the demands of secondary metabolism in the rubber tree may have driven the expansion of the *WOX* gene family, while the life history strategy of annual herb *E. hirta*, which favors rapid growth and reproduction, may reduce dependence on certain developmental regulatory genes. Previous phylogenetic classifications divided WOX proteins into three major clades [28,30,31,33]. The phylogenetic tree revealed conserved evolutionary patterns, with both *H. brasiliensis* and *E. hirta* containing four members in the ancient clade. Previous studies have shown that some members participate in lateral root development and floral organ formation (e.g., AtWOX13) in the ancient clade, while the Intermediate Clade plays a conserved role in root organogenesis. For example, IC-WOX proteins in ferns are expressed in root founder cells of adventitious and lateral roots, while seed plant IC-WOX subgroups (e.g., WOX11 and WOX8/9) are involved in root or shoot formation [8]. In this analysis, *H. brasiliensis* and *E. hirta* displayed differential retention in the Intermediate Clade (four in *H. brasiliensis* and three in *E. hirta*). Strikingly, the WUS clade showed the most dramatic expansion, containing 13 and seven members in *H. brasiliensis* and *E. hirta*, respectively. Previous studies have demonstrated that the core members of the WUS clade (e.g., WUS and WOX5) maintain stem-cell homeostasis in apical shoot meristems and apical root meristems, which are critical for post-embryonic plant development [8]. Additionally, a phylogenetic analysis of the 14 EhWOX proteins consistently resolved them into three characteristic clades (Figure 2B). These findings suggest functional divergence among WOX subfamilies, with WUS-clade genes potentially playing enhanced roles in species-specific secondary metabolism (e.g., latex biosynthesis), while ancient-clade members likely maintain conserved functions in fundamental developmental processes.

The TF motifs serve as core regulatory elements that are crucial for regulating gene expression. Although the homeodomain of WOX proteins is highly conserved, substantial variations exist in other motifs [34]. EhWOX motif prediction revealed that motif1 and motif3 represent two segments of the homeodomain and constitute characteristic signatures of the WOX family, being present in all EhWOX proteins, consistent with reports of motif1 and motif2 in four Euphorbiaceae species [24,35,36,37]. Notably, motif2 was exclusively predicted in the four proteins of the ancient clade, corresponding to motif4, which is uniquely present in the four Euphorbiaceae species [24]. Remarkably, motif5 was only detected in two proteins of the ancient clade, suggesting the significance of motif2 and motif5 in plant development and highlighting the evolutionary conservation of ancient-clade proteins (Figure 2B). These predicted motif distribution patterns demonstrate considerable divergence among different clades, implying functional differentiation across WOX families. WOX proteins belong to the homeobox TF superfamily, characterized by a conserved 60-amino-acid homeodomain that forms a helix–loop–helix–turn–helix structure for specific DNA binding (Figure 2E) [28,38]. In this study, 11 EhWOX proteins were found to belong to the canonical homeodomain family, while 3 (EhWOX10, EhWOX9, and EhWOX1) showing structural variations were found to belong to the Homeodomain superfamily (Figure 2C). In the conserved sequence alignment, compared with other members, EhWOX10, EhWOX9, and EhWOX1 exhibit amino acid variations; for instance, EhWOX1 shows a Glu–to–Thr substitution at position 9 in Helix1. These variations may represent potential markers of functional innovation or Euphorbiaceous-specific subfamily differentiation. Gene structure prediction further revealed that the exon–intron organization of *WOX* families in *E. hirta* showed high similarity to those in four Euphorbiaceae species, typically containing two to four CDS regions (Figure 2D) [24], highlighting both the high degree of structural conservation in *WOX* genes throughout evolution and their critical function in plant growth and development. Through chromosomal localization and synteny analysis in *E. hirta*, three syntenic gene pairs were identified, whose evolution likely resulted from tandem and segmental duplication events (Figure 3A, Supplementary Table S2). Additionally, comparative genomic analysis was performed to investigate the syntenic relationships of *WOX* genes among *E. hirta*, *H. brasiliensis*, *T. kok-saghyz*, and *M. esculenta*, reflecting phylogenetic relationships based on gene-family evolution. The predicted results indicate that orthologous genes in plants have undergone duplication or loss during evolution, leading to differential syntenic conservation of *WOX* genes among species.

Cis-acting elements act as indispensable molecular switches in gene expression [39,40,41]. In predicted elements, development-related elements were the most abundant, including light-responsive elements (e.g., Box 4, G-box, and GT1 motif). All promoters of *EhWOX* gene contained Box 4, with the *EhWOX10* gene harboring the highest number (nine), indicating extensive involvement of *WOX* genes in light-regulated development. Furthermore, hormone-related elements (e.g., ABA and JA-responsive elements) were present in *EhWOX* promoters, as corroborated between hormones and malate in regulating *MdWOX11*-mediated development in apple [42]. Additionally, stress-related elements (e.g., ARE, O2-site, and CAT-box) were identified in the *EhWOX* family. Studies have demonstrated that *WOX* genes widely participate in abiotic stress responses, for example, *OsWOX5* and *OsWOX10* in rice, along with overexpression of *JcWOX5* from *J. curcas*, enhancing drought tolerance [43,44,45], while *OsWOX11* in rice and *PagWOX11/12a* in poplar respond to salt and nutrient stress. Collectively, these results underscore the crucial roles of the *WOX* family in plant development and abiotic stress adaptation, with evolutionary conservation further emphasizing their significance. Moreover, we predicted intergenic regulatory relationships based on transcription factor binding motifs (TFBMs). Although prediction indicates low functional overlap between TF binding and gene regulation, alongside the non-linear relationship between motif binding and gene regulation [46], the TFBM-based regulatory network established here provides a foundational framework for subsequent research.

Gene function is closely linked to tissue-specific expression patterns, and different members of the *WOX* gene family are expressed and functional in diverse plant tissues, including roots, stems, leaves, flowers, and meristems [8,47]. In this study, RNA-seq expression analysis revealed that *EhWOX7* and *EhWOX14* are highly expressed in roots, stems, leaves, flowers, and latex (Figure 6A), with RT-qPCR validating the accuracy of RNA-seq data in stems and leaves (Figure 6C,D). Furthermore, phylogenetic analysis placed *EhWOX14* within the ancient clade (Figure 2A), suggesting functional conservation of this gene in *E. hirta*. RNA-seq results indicated that seven genes were highly expressed in flowers and four in roots, consistent with expression patterns reported in *Jatropha curcas* [24]. Two *EhWOX* genes were expressed in latex and leaves, respectively, implying their functions in these tissues. In stems, *EhWOX4*, *EhWOX5*, and *EhWOX6* were expressed. Phylogenetically, *EhWOX5* and *EhWOX6* clustered with *A. thaliana AtWOX14* in the ancient clade, while *EhWOX4* clustered with *AtWOX4* in the WUS clade (Figure 2A). Furthermore, RT-qPCR confirmed *EhWOX4*, *EhWOX5* and *EhWOX6* high expression in stems (Figure 6D). Additionally, as TFs, EhWOX4, EhWOX5 and EhWOX6 were predicted and verified to localize to the nucleus (Table 1, Figure 7A), and yeast assays confirmed their transactivation activity (Figure 7B). Previous research has shown that *AtWOX14* promotes cell differentiation in the cambium region to regulate stem development in *A. thaliana* [22] and *WOX4* regulates stem development [18,19,20]. The expression profiles and bioinformatics analysis of EhWOX4, EhWOX5, and EhWOX6 suggest their potential involvement in stem development, though further validation is required. Notably, *E. hirta* possesses advantageous traits for model plant studies, including a compact stature, short life cycle, and adaptability to controlled cultivation (Figure 6B), making it an ideal system for the study of stem development. Consequently, the validation of highly expressed genes in the stems of this species provides significant application value.

This study represents the first systematic identification of 14 *EhWOX* genes in *E. hirta*, along with comprehensive characterization of their evolutionary conservation and expression patterns. Our integrative analyses suggest potential roles for EhWOXs in stem development, thereby establishing a foundation for functional studies of *EhWOX* genes in *E. hirta*. In the future, investigations employing gene knockout, overexpression, spatial expression analysis of stems, and chromatin immunoprecipitation to identify downstream targets could validate the proposed regulatory mechanisms of *EhWOX* genes in stem development. Therefore, such a study would provide a systematic roadmap for functional studies of the WOX family and highlight the utility of *E. hirta* as a model system for investigation of laticifer development.

## 4. Experimental Procedures

### 4.1. Experimental Materials

The wild-type *E. hirta* plants used in this study were provided by the Chinese Academy of Tropical Agricultural Sciences, located in Sanya City, Hainan Province, China (18°15′16.9″ N, 109°30′27.5″ E). For RT-qPCR analysis, *E. hirta* plants were cultivated under controlled growth-chamber conditions at 28 °C with 16 h of light (100 μmol m^−2^ s^−1^) followed by 8 h of darkness for 40 days. Then, stems and leaves from uniformly grown plants at three distinct developmental positions (S1–3 for stems; L1–L3 for leaves) were collected for RNA extraction and subsequent RT-qPCR analysis. *N. benthamiana* plants were used for subcellular localization assays. All plant materials were maintained under standardized conditions prior to experimental use to ensure consistency in physiological status.

### 4.2. Identification and Physicochemical Analysis of EhWOX Genes

The chromosome-level genome assembly of *E. hirta* and corresponding annotation files (GFF3) were provided by the Chinese Academy of Tropical Agricultural Sciences (unpublished). To identify *WOX* family members in *E. hirta*, we employed a dual approach: a Hidden Markov Model (HMM) search against the genome protein sequences using the homeodomain (http://pfam.xfam.org/, Pfam: PF00046, accessed on 15 May 2025) as a query with an E-value cutoff of 0.01 and a local BLASTP (BLAST+ v2.14.0) analysis using *A. thaliana* WOX protein sequences as queries with a stringent E-value threshold of <1 × 10^−10^. Candidate *EhWOX* genes were identified and named according to their chromosomal order. Subsequently, their physicochemical properties were analyzed using ExPASy ProtParam (https://web.expasy.org/protparam/, accessed on 17 May 2025). Protein-domain architecture was verified using the NCBI Conserved Domain Database (https://www.ncbi.nlm.nih.gov/Structure/bwrpsb/bwrpsb.cgi, accessed on 15 May 2025), while subcellular localization was predicted using WoLF PSORT (https://wolfpsort.hgc.jp/, accessed on 20 May 2025).

### 4.3. Phylogenetic Analysis of WOX Genes

To investigate the evolutionary relationships of *WOX* genes across species, we first analyzed the divergence times of 20 species, including *E. hirta*, *H. brasiliensis*, and *A. thaliana*, using the TimeTree web resource (TimeTree: The Timescale of Life, https://timetree.org/, accessed on 23 May 2025) while simultaneously counting the number of *WOX* family members in each species. For phylogenetic reconstruction of *WOXs* from eight selected species (Eh: *Euphorbia hirta*; WCJ: *Euphorbia lathyrism*; Ja: *Jatropha curcas*; Me: *Manihot esculenta*; Rc: *Ricinus communis*; rna-Hb: *Hevea brasiliensis*; Ptr: *Populus tomentosa*; At: *Arabidopsis thaliana*), we performed multiple sequence alignment of full-length protein sequences using MUSCLE (Version 5) with default parameters. The resulting alignment was subsequently refined using trimAL to remove poorly aligned regions. Maximum likelihood (ML) phylogenetic trees were constructed using IQ-TREE with 5000 ultrafast bootstraps replicates (default parameters) to assess node support in TBtools (v2.331) [48], and the appropriate model was selected utilizing the ModelFinder method. The JTTDCMut+G4 model was chosen according to the Bayesian Information Criterion (BIC). The same analytical pipeline was applied to construct the phylogenetic tree of *E. hirta* EhWOX family members, and the JTT+G4 model was used. All phylogenetic trees were visualized and annotated using the iTOL platform (https://itol.embl.de/help.cgi, accessed on 20 June 2025).

### 4.4. Structural Analysis of EhWOX Genes

The chromosomal localization and gene density distribution of the *EhWOX* gene family were visualized using the “Gene distribution Visualize” function in TBtools software with the input GTF/GFF annotation file. For conserved motif analysis, we employed the MEME suite (http://meme-suite.org/tools/meme, accessed on 27 May 2025) with the following parameters: number of motifs set to 5 and site distribution option configured as Zero or One Occurrence Per Sequence (zoops). The Conserved Homeodomain Domain prediction tool is the NCBI Conserved Domain Database tool (https://www.ncbi.nlm.nih.gov/Structure/cdd/wrpsb.cgi, accessed on 28 May 2025) with a cutoff of 0.01. The resulting motif patterns were subsequently integrated with phylogenetic relationships, a conserved homeodomain architecture, and exon–intron organization using the “Gene Structure View (Advanced)” plugin in TBtools, which enabled comprehensive visualization of these multi-dimensional structural features in a unified graphical representation.

### 4.5. Synteny Analysis

Intra-species synteny analysis of *EhWOX* genes in *E. hirta* was performed using the “One Step MCScanX” function in TBtools (v2.331) with default parameters (1 × 10^−10^), and the results were visualized, along with gene density and GC content data, using the “Advanced Circos” tool. For inter-species synteny analysis, the *H. brasiliensis* genome and GFF3 files, the *T. kok-saghyz* genome and GFF3 files downloaded from NCBI (https://www.ncbi.nlm.nih.gov, accessed on 5 June 2025), and the *M. esculenta* genome and GFF3 files obtained from Ensembl Plants (Ensembl Plants, accessed on 6 June 2025) were utilized. Comparative synteny analysis between these three species and *E. hirta* was conducted using the “One Step MCScanX” pipeline in TBtools with default settings, followed by comprehensive visualization of the syntenic relationships [49].

### 4.6. Analysis of Cis-Acting Elements of EhWOX Promoters

The 2000 bp upstream sequences of all identified *EhWOX* genes were extracted as putative promoter regions using the Gtf/Gff3 Sequences Extract function in TBtools software. These promoter sequences were subsequently analyzed using the PlantCARE database (http://bioinformatics.psb.ugent.be/webtools/plantcare/html/, accessed on 8 June 2025) to identify potential cis-regulatory elements. For downstream analysis, we filtered out common core promoter elements, including TATA-box, CAAT-box, and un-named elements, to focus on functionally relevant regulatory motifs. The distribution patterns of these cis-acting elements were visualized by mapping their positions along promoter sequences using the “Simple Biosequence Viewer” function in TBtools and quantitative analysis of element counts per gene presented as heatmaps generated with R statistical software (version 4.4.3).

### 4.7. Gene Ontology Enrichment and Regulatory Network Analysis

*E. hirta* GO annotation was performed using eggNOG-mapper (https://github.com/eggnogdb/eggnog-mapper, accessed on 11 June 2025) with the latest GO-basic.obo file (http://purl.obolibrary.org/obo/go/go-basic.obo, accessed on 11 June 2025) to functionally characterize *E. hirta* genes using TBtools (default parameters). The resulting GO terms were visualized using TBtools. To analyze the transcriptional regulatory network of *EhWOX* transcription factors, the “Plant TF Binding Motif Shift” function (default parameters) in TBtools was employed to retrieve binding motifs for all TFs in *E. hirta*, with data sourced from PlantTFDB (https://planttfdb.gao-lab.org/). Subsequently, the FIMO tool (default parameters) was utilized to predict the regulatory binding sites of 14 EhWOX TFs (Supplementary Table S4) in TBtools, and the potential regulatory-network target genes were visualized using R (version 4.4.3).

### 4.8. RNA-Seq and RT-qPCR Expression Analysis of EhWOXs

The RNA-Seq raw sequence data reported in this study have been submitted to the Genome Sequence Archive (Genomics, Proteomics and Bioinformatics 2025) at the National Genomics Data Center (Nucleic Acids Research 2025), China National Center for Bioinformation/Beijing Institute of Genomics, Chinese Academy of Sciences (https://ngdc.cncb.ac.cn/gsa, GSA accession number: CRA037671). Expression levels based on FPKM values were quantified and visualized as heatmaps using TBtools. For experimental validation, total RNA was extracted from *E. hirta* stems and leaves using an RNAprep Pure Plant Kit (Tiangen Biotech) following the manufacturer’s protocol. First-strand cDNA synthesis was performed with 1 μg total RNA as template using the HiScript II Q RT SuperMix reverse transcription kit (Vazyme Biotech). RT-qPCR reactions were carried out on a Light Cycler 480 II Real-Time PCR System (Roche, Basel, Switzerland) in 20 μL reaction volumes (10 μL AceQ qPCR SYBR Green Master Mix, 1 μL cDNA template, and 1 μL each of 10 μm forward and reverse gene-specific primers for 35 cycles) (primers in Supplementary Table S6). The actin gene (ACT1.2373) was used as internal reference for normalization, and relative gene expression levels were calculated using the 2^−ΔΔCt^ quantitative method. All expression data were statistically analyzed and visualized using GraphPad Prism software (version 10).

### 4.9. Subcellular Localization of EhWOX Proteins

Based on phylogenetic analysis and expression profiling, we selected proteins potentially involved in stem development for subcellular localization. The subcellular localization of *EhWOX4*, *EhWOX5*, and *EhWOX6* was determined by transiently expressing *pCAMBIA2300-EhWOX4-eGFP*, *pCAMBIA2300-EhWOX5-eGFP*, and *pCAMBIA2300-EhWOX6-eGFP* fusion constructs in *N. benthamiana*. First, the CDSs of the three *EhWOX* genes were amplified by PCR and subsequently cloned into the *pCAMBIA2300-35S-eGFP* vector [15]. The recombinant plasmids were verified by sequencing, then transformed into *Agrobacterium tumefaciens* strain *GV3101*. Bacterial suspensions (OD 600 ≥ 0.6) were infiltrated into *N. benthamiana* leaves using a needleless syringe. After infiltration, the plants were incubated in the dark for 48 h, and GFP fluorescence signals (488 nm) were observed and acquired using a confocal laser scanning microscope (Zeiss LSM800, Göttingen, Germany). Meanwhile, *OsWRKY70* was co-injected for concurrent expression as a fluorescence-based localization marker.

### 4.10. Yeast Autoactivation Assay for EhWOXs

A yeast assay was employed to determine whether the TF *EhWOXs* possessed autoactivation. The CDSs of *EhWOXs* were amplified by PCR and subsequently cloned into the *pGBKT7* vector to construct recombinant plasmids *pGBKT7–EhWOX*. These plasmids were then transformed into yeast strain *AH109*. The transformed yeast cells were plated on SD/-Trp medium and cultured at 30 °C for 2–3 days, after which single colonies were selected for further verification. Positive transformants were inoculated onto SD/-Trp or SD/-Trp/-AbA (AbA final concentration: 200 ng/mL) plates and cultured at 30 °C for 2–3 days to observe growth. Subsequently, X-α-gal (4 μg/mL) was spotted onto the yeast colonies, followed by incubation at 30 °C for 2–12 h to monitor the formation of blue colonies.

### 4.11. Statistical Analysis

Statistical significance was defined as *p* < 0.05, and analyses were performed using One-way ANOVA and Tukey’s multiple comparison test. Data visualization was performed using GraphPad Prism (v 9.0.0).

## 5. Conclusions

In this study, we systematically identified and characterized 14 *EhWOX* genes in *E. hirta*. Phylogenetic and structural analyses classified them into three conserved clades and revealed group-specific motifs. Expression profiling revealed tissue-specific patterns, with several genes showing stem-predominant expression that was validated by RT-qPCR. The nuclear localization and transcriptional autoactivation potential of stem-expressed members were confirmed. While RNA-seq data suggest some members may be expressed in latex-producing tissues, their specific cellular roles require future spatial and functional validation. Collectively, this comprehensive genomic and transcriptomic characterization establishes an essential foundation and identifies specific candidate genes for future functional studies on stem development and, potentially, laticifer biology in *E. hirta*.

## Figures and Tables

**Table 1:** Physicochemical properties of the WOX family.

Protein Name	Protein ID	Size (AA)	MW (kD)	pI	Instability Index	Aliphatic Index	Grand Average of Hydropathicity	Subcellular Localization
EhWOX1	evm.model.Chr1.2302	176	20.140	9.220	68.980	58.120	−0.835	nucl
EhWOX2	evm.model.Chr2.1103	370	42.360	7.700	56.300	52.540	−0.944	nucl
EhWOX3	evm.model.Chr2.3670	268	30.610	9.430	65.090	51.750	−1.057	nucl
EhWOX4	evm.model.Chr3.3752	218	24.850	9.430	53.160	68.850	−0.803	nucl
EhWOX5	evm.model.Chr3.4368	210	24.040	6.310	65.050	72.000	−0.665	nucl
EhWOX6	evm.model.Chr3.4371	210	24.010	5.970	66.690	73.860	−0.648	nucl
EhWOX7	evm.model.Chr4.1352	225	25.340	5.660	43.090	67.200	−0.903	nucl
EhWOX8	evm.model.Chr5.280	385	42.100	6.890	64.130	69.690	−0.475	nucl
EhWOX9	evm.model.Chr6.2213	257	28.430	5.260	55.960	68.250	−0.482	nucl
EhWOX10	evm.model.Chr6.2214	241	26.660	5.950	57.830	64.690	−0.488	nucl
EhWOX11	evm.model.Chr7.1283	170	19.900	9.550	74.230	53.940	−0.747	nucl
EhWOX12	evm.model.Chr7.1331	237	26.760	8.460	59.010	55.910	−0.832	nucl
EhWOX13	evm.model.Chr8.1626	177	20.660	9.830	53.350	63.900	−0.680	nucl
EhWOX14	evm.model.Chr9.592	248	27.660	5.150	45.600	65.690	−0.843	nucl

## Data Availability

All data can be found in the article or in the Supplementary Materials.

## Supplementary Materials

The following supporting information can be downloaded at: https://www.mdpi.com/article/10.3390/plants15030509/s1. Table S1: Sequences of EhWOX; Table S2: Duplication events of *EhWOX* genes; Table S3: GO terms of *EhWOX*; Table S4: Regulatory binding sites of 14 *EhWOX* transcription factors; Table S5: Expression values of *WOX* genes in different tissues based on RNA-seq; Table S6: Primers for experiment.
